# Endothelial specific prolyl hydroxylase domain-containing protein 2 deficiency attenuates aging-related obesity and exercise intolerance

**DOI:** 10.1007/s11357-024-01108-0

**Published:** 2024-03-11

**Authors:** Lihong Pan, Xiaochen He, Rui Xu, Umesh Bhattarai, Ziru Niu, Jussara do Carmo, Yuxiang Sun, Heng Zeng, John S. Clemmer, Jian-Xiong Chen, Yingjie Chen

**Affiliations:** 1 Department of Physiology and Biophysics, University of Mississippi Medical Center, School of Medicine, 2500 North State Street, Jackson, MS 39216 USA; 2 Department of Nutrition, Texas A&M University, College Station, TX USA; 3 Department of Pharmacology and Toxicology, University of Mississippi Medical Center, School of Medicine, Jackson, MS 39216 USA

**Keywords:** Obesity, Metabolism, Energy expenditure, Insulin resistance, Aging

## Abstract

Obesity and exercise intolerance greatly reduce the life quality of older people. Prolyl hydroxylase domain-containing protein 2 (PHD2) is an important enzyme in modulating hypoxia-inducible factor-alpha (HIF) protein. Using vascular endothelial cell-specific PHD2 gene knockout (PHD2 ECKO) mice, we investigated the role of endothelial PHD2 in aging-related obesity and exercise capacity. Briefly, PHD2 ECKO mice were obtained by crossing PHD2-floxed mice with VE-Cadherin (Cdh5)-Cre transgenic mice. The effect of PHD2 ECKO on obesity and exercise capacity in PHD2 ECKO mice and control PHD2^f/f^ mice were determined in young mice (6 to 7 months) and aged mice (16–18 months). We found that aged PHD2 ECKO mice, but not young mice, exhibited a lean phenotype, characterized by lower fat mass, and its ratio to lean weight, body weight, or tibial length, while their food uptake was not reduced compared with controls. Moreover, as compared with aged control mice, aged PHD2 ECKO mice exhibited increased oxygen consumption at rest and during exercise, and the maximum rate of oxygen consumption (VO_2_ max) during exercise. Furthermore, as compared with corresponding control mice, both young and aged PHD2 ECKO mice demonstrated improved glucose tolerance and lower insulin resistance. Together, these data demonstrate that inhibition of vascular endothelial PHD2 signaling significantly attenuates aging-related obesity, exercise intolerance, and glucose intolerance.

**Supplementary Information:**

The online version contains supplementary material available at 10.1007/s11357-024-01108-0.

## Introduction

Obesity and reduced exercise capacity or mobility disorder are major medical problems that greatly decrease the life quality of older populations. Obesity, reduced exercise capacity, and metabolic dysfunction also contribute to the development of various diseases during the aging process [1, 2].

Prolyl hydroxylase domain-containing protein 2 (PHD2), encoded by the EGLN1 gene, is a key enzyme responsible for mediating hypoxia-inducible factors (HIFs) that respond to decreases in oxygen bioavailability in the cellular environment. HIFs are heterodimeric proteins consisting of an O2-sensitive HIF-1α, HIF-2α, or HIF-3α subunit and a constitutively expressed HIF-1β subunit. PHD2 knockdown or inhibition is known to induce HIF activity in most cell types [3, 4]. Hypoxia, a condition characterized by reduced oxygen levels, has been shown to decrease body weight [5–7]. Although the mechanism behind hypoxia-induced suppression of fatty acid synthesis and reduction in fat mass is not fully understood [8, 9], the role of the oxygen-sensing pathway in metabolism has garnered considerable attention. This pathway involves both HIFs and PHDs [10]. PHDs catalyze the oxygen-dependent hydroxylation of specific proline residues in HIF-1α, HIF-2α, or HIF-3α, leading to its degradation. Hypoxia diminishes PHD activity, resulting in increased HIF expression and the activation of target genes involved in metabolism [11]. HIF-α has been implicated in the attenuation of obesity, fatty liver disease, and type 2 diabetes [12, 13], as well as in regulating energy balance and metabolism in the hypothalamus [14–17]. Furthermore, loss of skeletal muscle HIF-1α has resulted in metabolic shift away from glycolysis and toward oxidation, and increased sensitivity to exercise-induced muscle injury in mice [18]. Moreover, studies have demonstrated that PHD2 plays a critical role in diet-induced obesity and glucose intolerance [19]. While HIF may hold therapeutic potential, direct manipulation of HIF in vivo is challenging. In contrast, PHDs represent an ideal target for modulating HIF levels, and several PHD inhibitors have been developed.

In this study, we investigated the effects of endothelial-specific PHD2 deficiency on obesity and exercise tolerance in young adult and aged mice. We hypothesized that the deletion of endothelial PHD2 will alleviate obesity and enhance exercise capacity in aged mice.

## Materials and methods

### Animals

The experimental procedures conducted in this study adhered to the guidelines outlined in the National Institutes of Health Guide for the Care and Use of Laboratory Animals. The research protocol was approved by the Institutional Animal Care and Use Committee of the University of Mississippi Medical Center. Vascular endothelial cell-specific PHD2 knockout mice (**PHD2 ECKO**) mice were generated using PHD2 flox/flox (PHD2^f/f^) mice [20] and VE-Cadherin (Cdh5)-Cre transgenic mice (Jackson Lab, # Strain #:**006137**) [21] as previously described [22]. PHD2^f/f^ mice were used as the control mice for PHD2 ECKO mice. Young male PHD2 ECKO mice (n = 9), young male PHD2^f/f^ mice (n = 9), aged male PHD2 ECKO mice (n = 19), and aged male PHD2^f/f^ mice (n = 13) were used for the study. Mice were fed ad libitum with normal chow throughout their lives, and experimental tests were conducted in mice at young mice (6 to 7 months), and aged mice (16 to 18 months). Samples were collected after intraperitoneal injection of 100 mg/kg Ketamine and 10 mg/kg Xylazine.

### Glucose tolerance test and insulin tolerance test

Mice were fasted for 6-h prior to the glucose tolerance test as previously described [23]. After baseline glucose measurement, the mice were intraperitoneally injected with a bolus of glucose (1 mg/g of lean body weight). Blood glucose levels were measured 0, 15, 30, 60, 90 and 120 min after the injection. For the insulin tolerance test, the mice received an intraperitoneal injection of a bolus of insulin (0.75 IU/kg of lean body weight). Blood samples were collected from the tail vein and measured 0, 15, 30, 45, 60 and 90 min after the injection. blood glucose concentrations were measured using the Glutest Every kit (Sanwa Kagaku Kenkyusho, Japan).

### Tissue weight and body composition analysis

The fat and lean masses content from PHD2 ECKO and control mice were assessed by using a magnetic resonance imaging system (EchoMRI-900TM, Echo Medical System, Houston, TX). Subsequently, the mice were euthanized and measurements of body weight, tibia length, white adipose tissue weight, and liver weight were taken.

### Energy expenditure measurements in aged mice

Aged PHD2 knockout (KO) mice and PHD2f/f control mice were individually housed in metabolic cages (Promethion Metabolic and Behavioral System, Sable Systems International, Las Vegas, NV) to assess oxygen consumption, food intake, and energy expenditure (EE). These mice were allowed to acclimate to the new environment for 3 days before recording data for 3 consecutive days.

### Food intake measurement in young mice

Daily food intake and body weight were also measured in young PHD2 ECKO and age-matched control mice over 3 days period.

### Graded maximal exercise capacity test

Mice were acclimated on the exercise treadmill 1 time /day for 3 consecutive days then followed by a graded maximal exercise capacity test as previously described by Petrosino et al [24]. Briefly, the graded maximal exercise capacity test protocol was performed by placing the mice on the treadmill (Columbus Instruments, OH) at 5° inclination. The treadmill speed was then gradually increased as follows (speed, duration, inclination): 9 m/minute, 2 min, 5°; 12 m/minute, 2 min, 10°; 15 m/minute, 2 min, 15°; 18 m/minute, 1 min, 15°; 21 m/minute, 1 min, 15°; 23 m/minute, 1 min, 15°; and an increase in speed 1 m/minute for every minute thereafter. Exhaustion is defined as the point at which mice maintained continuous contact with the shock grid for 10 s without the intent of returning to the belt to continue to run. VO_2_, VCO_2_, Δ VO2, respiratory exchange ratio (RER), and heat production were recorded every 30 s by the Oxymax software (Columbus Instruments, OH) and normalized to body weight. VO_2_max was determined by the peak oxygen consumption reached during this test when RER was ≥ 1.0. The Δ VO2 was determined by VO2max subtract by VO2 at rest.

### Histological analysis

The adipose tissues and liver were fixed in a 10% neutral buffered formaldehyde solution overnight and subsequently embedded in paraffin. Paraffin sections were then subjected to hematoxylin and eosin (H&E) staining. Ten images of H&E staining sections were captured from each sample.

### Statistical analysis

The results are presented as means ± SE. To evaluate significant differences between the two groups, an unpaired Student's t-test was employed. For comparisons between two groups over time, two-way ANOVA with repeated measures was conducted, followed by Bonferroni post hoc multiple comparisons. Statistical significance was determined at a level of P < 0.05. Additionally, three-way ANOVA tests were utilized to assess the effects of different variables (such as age and strain) and their interactions on body fat/lean mass, body weight, food intake, energy expenditure, and motor activity.

## Results

### PHD2 Deficiency in endothelial cells abolished age-related obesity in Mice

**Fig. 1:**
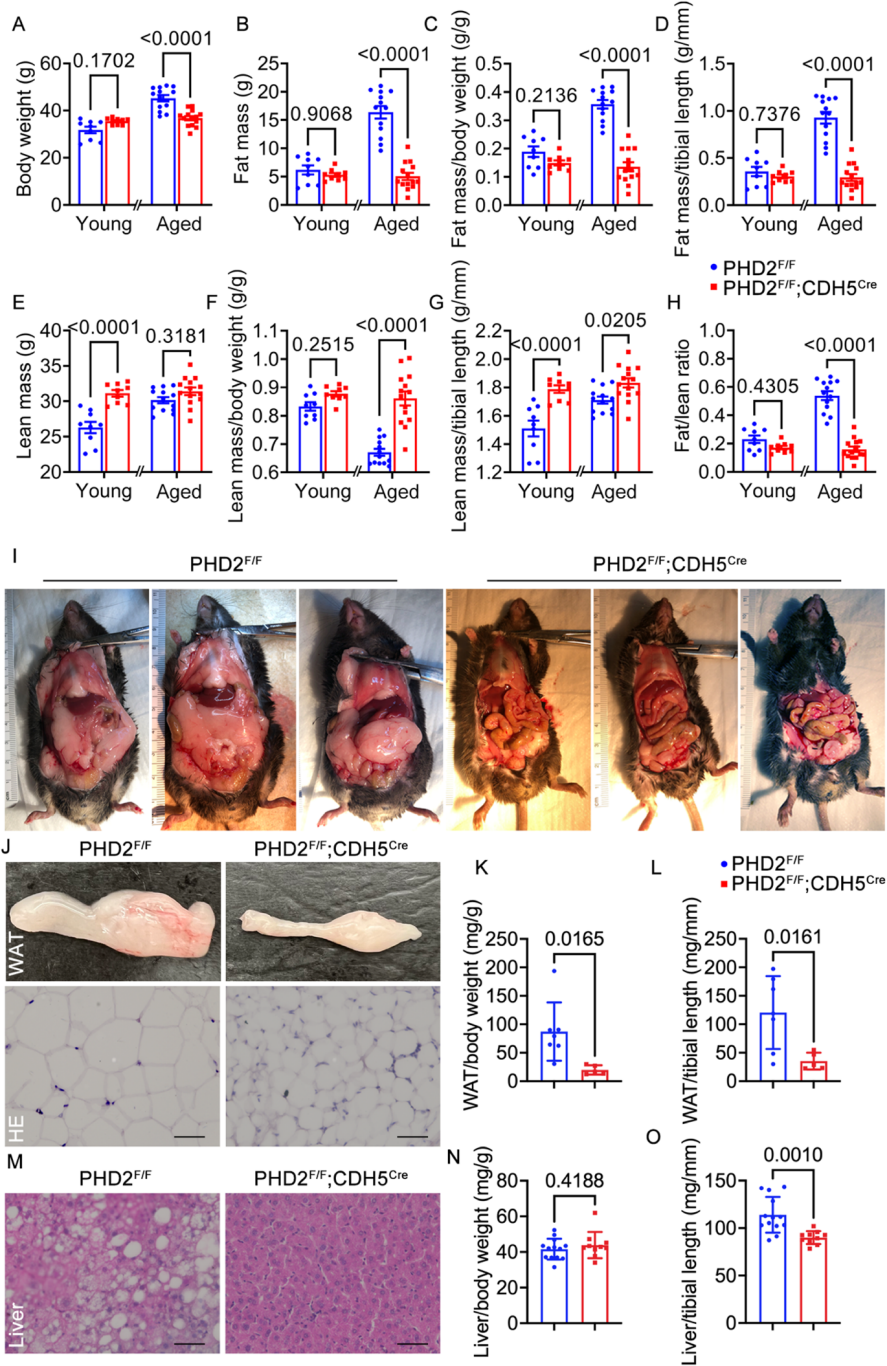
PHD2 ECKO mice are protected from aging-dependent obesity. **A**, PHD2 ECKO mice, and control mice were allowed to age in the laboratory animal center, and their body weight was monitored, two-way ANOVA, *p < 0.05. **B-D,** Fat mass, fat mass normalized to body weight, fat mass normalized to the tibial length of both young and aged PHD2 ECKO mice and control PHD2^f/f^ mice, unpaired 2-tailed t-test. **E–G,** Lean mass, lean mass normalized to body weight, lean mass normalized to the tibial length of PHD2 ECKO mice and control PHD2^f/f^ mice, unpaired 2-tailed t-test. **H,** fat/ lean ratio of PHD2 ECKO mice and control PHD2^f/f^ mice. **I,** representative image of aged PHD2 ECKO mice and aged control PHD2^f/f^ mice. **J,** representative image and hematoxylin and eosin staining of white adipose tissue (WAT) from aged PHD2 ECKO mice and aged control PHD2^f/f^ mice. Scale bar, 50 μm. **K,** quantification of WAT weight normalized to body weight. **L,** quantification of WAT weight normalized to tibial length, unpaired 2-tailed t-test. **M,** representative hematoxylin and eosin staining of liver tissue from aged PHD2 ECKO mice and aged control PHD2^f/f^ mice. Scale bar, 50 μm. **N,** quantification of WAT weight normalized to body weight. **O,** quantification of liver weight normalized to tibial length, unpaired 2-tailed t-test. N = 9, 9, 13, 14

PHD2 ECKO mice showed a remarkable prevention of aging-related obesity, with no significant differences in body weight, fat mass, or fat mass ratios compared to control group in young mice. In contrast, aged control mice exhibited significant increases in body weight, fat mass, and the ratio of fat mass to bodyweight or lean mass (Fig. 1A-D). While PHD2 ECKO mice exhibited increased lean mass and lean mass ratios to tibial length in youth, there were no discernible differences in terms of lean mass ratios to body weight and fat mass ratios to lean mass (Fig. 1E-H). We also observed significantly increased lean mass ratios to body weight and fat mass ratios to lean mass (Fig. 1F, H). Furthermore, aged PHD2 ECKO mice had substantially reduced fat mass, smaller adipocytes, and lower white adipose tissue, emphasizing the role of PHD2 in age-related obesity prevention (Fig. 1I-L). Additionally, liver fat content and weight were lower in aged PHD2 ECKO mice (Fig. 1M-O). Overall, PHD2 deficiency demonstrated a pivotal role in mitigating age-related obesity. We also reconfirmed that PHD2 expression was reduced in vascular endothelial cells in PHD2 ECKO mice, while HIF1α protein expression was significantly increased in heart and lung tissues in PHD2 ECKO mice (Supplemental Fig. 1).

### PHD2 Deficiency increased the overall energy expenditure without affecting the food uptake in aged mice

**Fig. 2:**
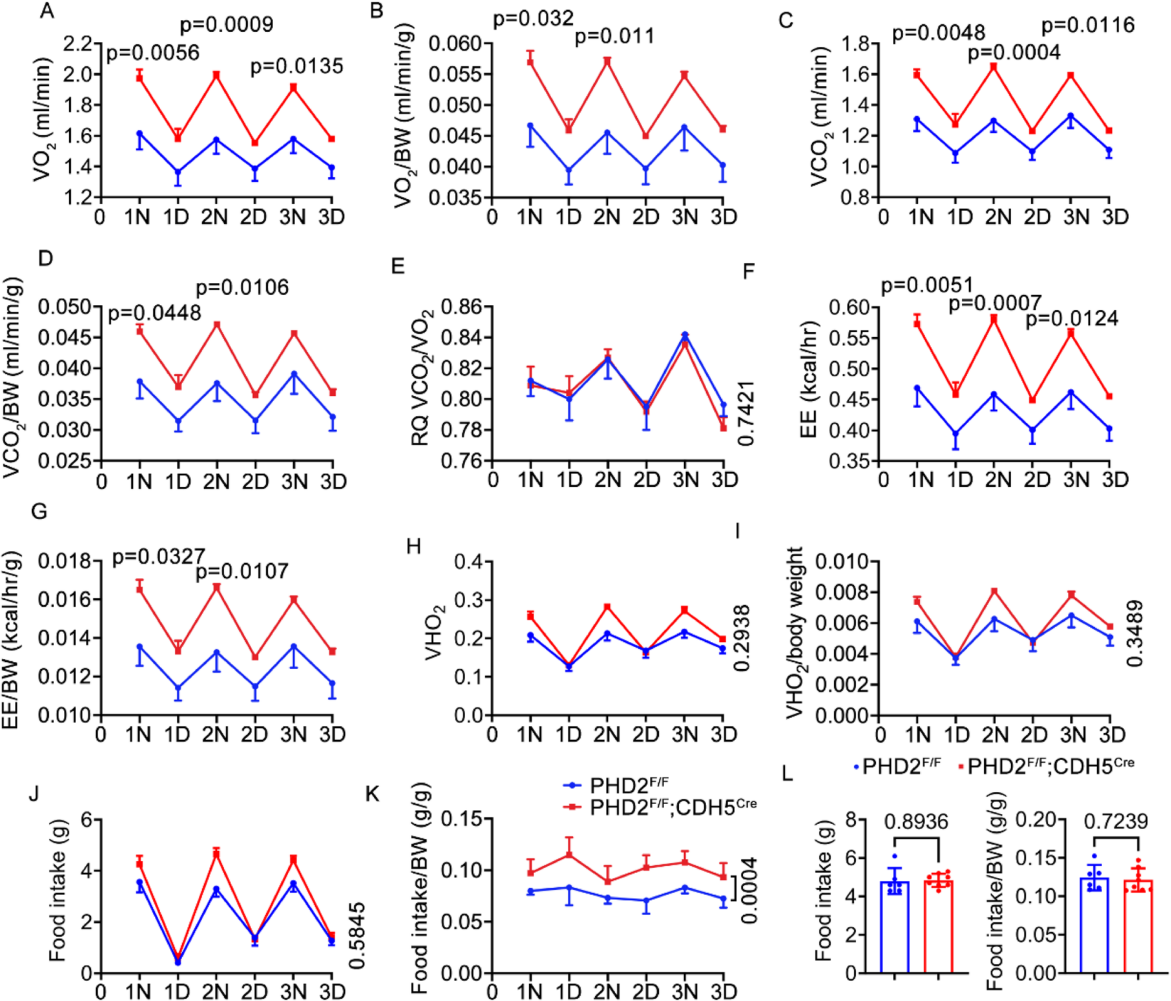
Energy Expenditure in Aged Mice. **A**, **B**, the daytime and nighttime oxygen consumption in PHD2 ECKO and control mice, both normalized to body weight (BW) and without normalization. **C, D,** daytime and nighttime carbon dioxide production in PHD2 ECKO and control mice, both normalized to body weight (BW) and without normalization. **E,** daytime and nighttime respiratory quotient (RQ) on PHD2 ECKO and control mice. **F, G,** daytime and nighttime energy expenditure (EE) in PHD2 ECKO and control mice, both normalized to body weight (BW) and without normalization. **H, I,** daytime and nighttime water intake in PHD2 ECKO and control mice, both normalized to body weight (BW) and without normalization. **J, K,** daytime and nighttime food intake in PHD2 ECKO and control mice, both normalized to body weight (BW) and without normalization. **L,** food intake with or without normalized to BW in young PHD2 ECKO and young PHD2^f/f^ control mice, unpaired 2-tailed *t-test*, n = 6,7

To gain further insights into the mechanism of age-related obesity in PHD2 ECKO mice, we conducted metabolic rate measurements using indirect calorimetry in metabolic chambers. Interestingly, aged PHD2 ECKO mice exhibited higher oxygen consumption than the PHD2^f/f^ control mice (Fig. 2A, B). We also observed an increase in carbon dioxide production in aged PHD2 ECKO mice as compared with the aged PHD2^f/f^ control mice (Figs. 2C, 2D). However, the respiratory quotient (RQ) was not significantly different between the aged PHD2 ECKO and PHD2^f/f^ control mice (Fig. 2E).

PHD2 ECKO mice consistently exhibited significantly higher daytime energy expenditure (EE), regardless of whether normalized or not by body weight (Fig. 2F, G). There were no notable differences in water intake between the two groups (Fig. 2H and I). Furthermore, we did not observe differences in food intake at different time points (Fig. 2J, K). Interestingly, total food uptake per mouse was unchanged, but the food uptake after normalized to body weight was significantly increased in PHD2 ECKO mice (Fig. 2K). However, the food intake of young PHD2 ECKO mice was unchanged as compared with young PHD2^f/f^ control mice (Fig. 2L). These findings indicate that the reduced fat mass in aged PHD2 ECKO mice is mainly an outcome of increased energy expenditure but not an outcome of reduced food consumption.

### PHD2 deficiency preserved metabolic and exercise capacity in aged mice

**Fig. 3:**
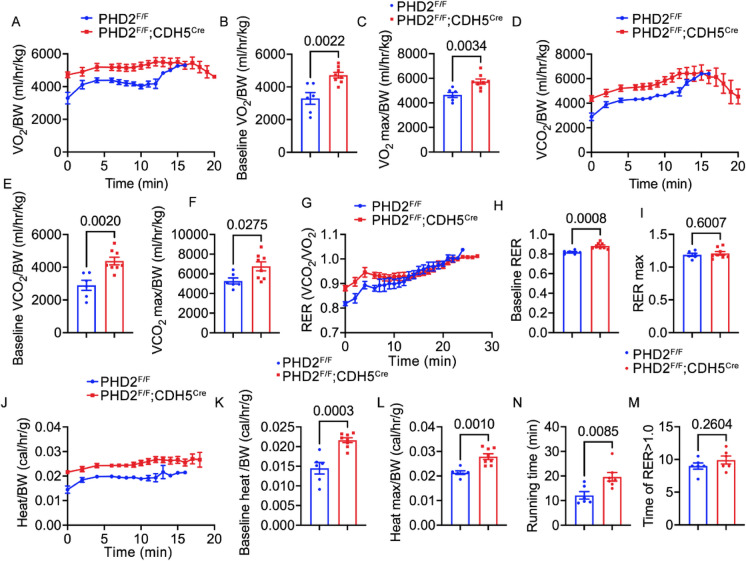
The respiratory exchange ratio (RER) during treadmill exercise in aged mice. **A-M,** PHD2 ECKO mice and control groups were run on the treadmill and their metabolism and running duration were monitored. **A,** O_2_ consumption. **B,** quantification of baseline O_2_ consumption. **C,** quantification of maximum O_2_ consumption. **D,** CO_2_ production. **E,** quantification of baseline CO_2_ production. **F,** quantification of maximum CO_2_ production. **G,** RER. **H,** quantification of baseline RER. **I,** quantification of maximum RER. **J,** heat production. **K,** quantification of baseline heat production. **L,** quantification of maximum heat production. **N,** quantification of running time for each group. **M,** quantification of the maximum running speed. Unpaired 2-tailed t-test, n = 6, 8

Since we observed an increased in daily oxygen consumption in aged PHD2 ECKO mice, we further assessed VO_2_ consumption, VCO_2_ production, and endurance exercise capacity, utilizing a graded maximal exercise capacity test protocol in both aged and young PHD2 ECKO mice. During the test, as the speed increased, some mice reached their endurance limit and were removed from the analysis. In our study, once 25% of the mice dropped out, their VO_2_ and VCO_2_ recordings were excluded from the final presentation. Consistent with the findings from metabolic chambers, we found increased VO_2_ consumption in the PHD2 ECKO group, both at baseline and maximum levels (Fig. 3A-C). No significant differences were observed in the ΔVO2 (Supplemental Fig. 2A). Similarly, we observed elevated VCO_2_ production in the PHD2 ECKO group under both baseline and maximum production conditions (Fig. 3D-F). Regarding nutrient utilization, as indicated by the respiratory exchange ratio (RER), we observed a higher baseline RER in the PHD2 ECKO group but no significant difference in the maximum condition (Fig. 3G-I). Furthermore, we detected increased heat production in both baseline and maximum conditions within the PHD2 ECKO group (Fig. 3J-L). Notably, the PHD2 ECKO group demonstrated a significantly longer running time during the exercise protocol, indicating enhanced endurance (Fig. 3N). Moreover, the maximum running speed in PHD2 ECKO mice was also significantly higher (Fig. 3M). However, the life span of aged PHD2 ECKO mice was reduced as compared with aged control mice, as 5 out of 19 aged PHD2 ECKO died during the study.

Together, these findings showed that PHD2 ECKO significantly abolished the aging-induced reduction of exercise capacity and the reduction of aerobic capacity, but these changes failed to improve the life span of PHD2 ECKO mice.

### Minimal influence of PHD2 ECKO on metabolic responses to treadmill exercise in young mice

**Fig. 4:**
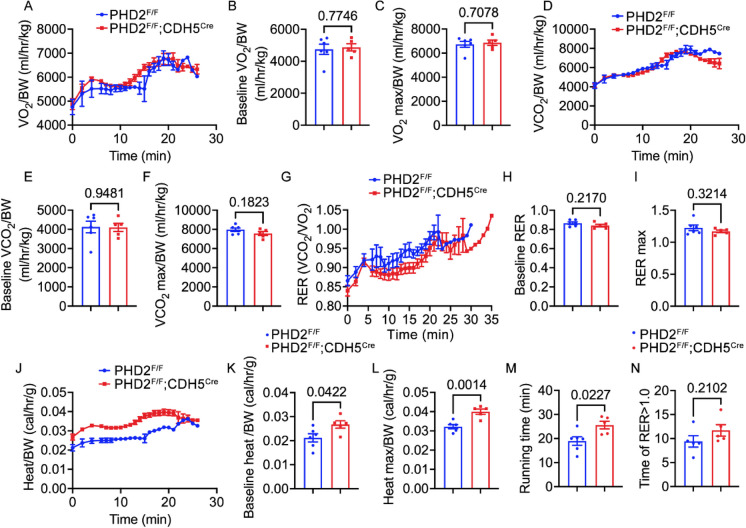
The respiratory exchange ratio (RER) during treadmill exercise in young mice. **A-M,** PHD2 ECKO mice and control groups were run on the treadmill and their metabolism and running duration were monitored. **A,** O_2_ consumption. **B,** quantification of baseline O_2_ consumption. **C,** quantification of maximum O_2_ consumption. **D,** CO_2_ production. **E,** quantification of baseline CO_2_ production. **F,** quantification of maximum CO_2_ production. **G,** RER. **H,** quantification of baseline RER. **I,** quantification of maximum RER. **J,** heat production. **K,** quantification of baseline heat production. **L,** quantification of maximum heat production. **M,** quantification of the running time for each group. **N,** quantification of the running duration of RER over 1. Unpaired 2-tailed t-test, n = 6, 5

Baseline VO_2_ and VO_2_ max consumption were measured in PHD2 ECKO mice and control mice, revealing no significant differences between the two groups (Fig. 4A-C, Supplemental Fig. 2A). Similarly, we assessed VCO_2_ production in both groups and obtained comparable results (Fig. 4D-F). Furthermore, nutrient utilization was analyzed using indirect calorimetry, where measurements of VCO_2_ production and VO_2_ consumption were employed to calculate the respiratory exchange ratio (RER). Consistent with previous findings, no notable differences were observed between young PHD2 ECKO and control groups (Fig. 4G-I). Intriguingly, we investigated heat production in the two groups and observed a significantly higher heat production in PHD2 ECKO mice, both at baseline and during maximum heat production (Fig. 4J-L). Additionally, the PHD2 ECKO group exhibited a significantly longer running time (Fig. 4M). However, the duration of RER above 1 remained similar between the two groups (Fig. 4N). These findings collectively demonstrate that deficiency in young mice has minimal impact on metabolic responses, including VCO_2_ production, VO_2_ consumption, and nutrient utilization as indicated by RER. Notably, PHD2 ECKO mice exhibit increased heat production and enhanced exercise endurance, suggesting potential effects on thermogenesis and physical performance.

### PHD2 deficiency improved glucose tolerance and insulin sensitivity in both young and aged mice

**Fig. 5:**
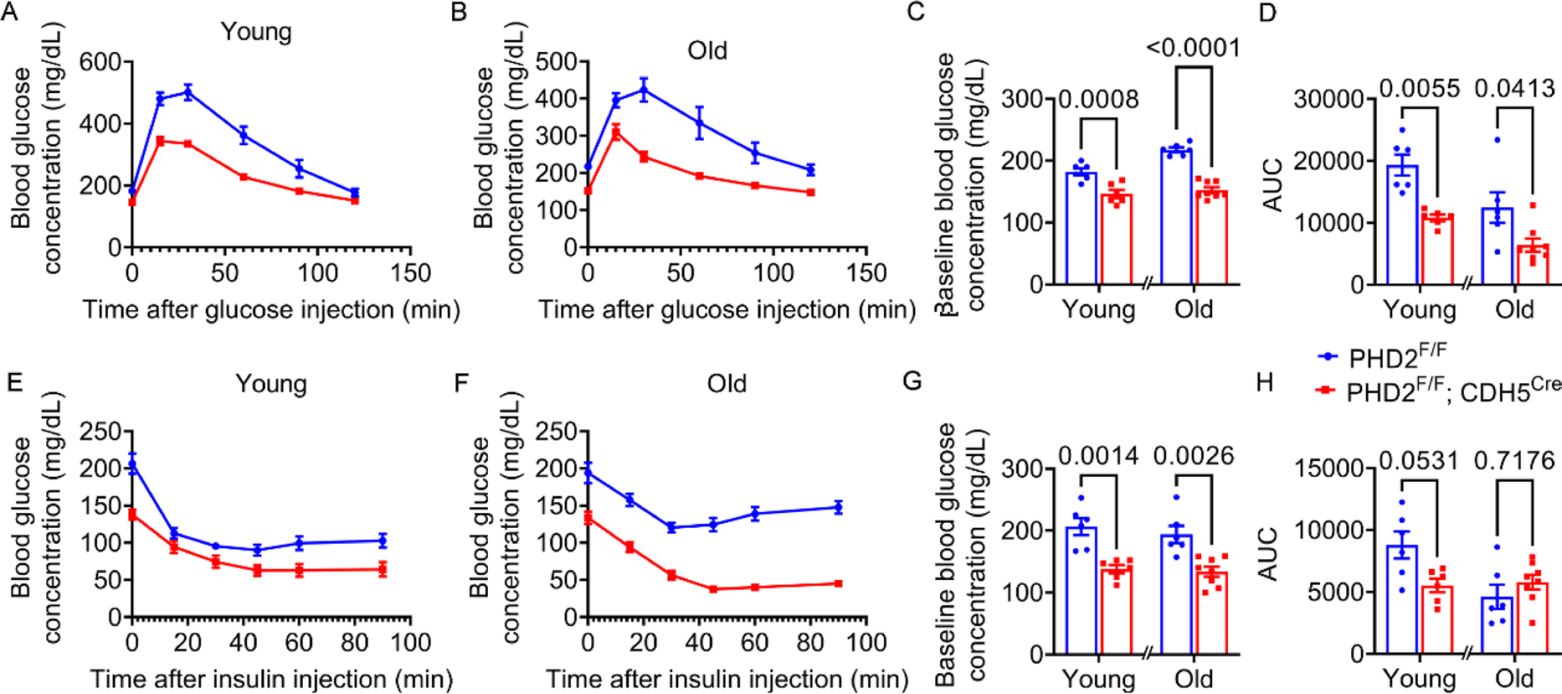
PHD2 ECKO mice show improved glycemic control. **A, B,** Intraperitoneal glucose tolerance test performed on PHD2 ECKO and control young and aged mice. Glucose was measured from blood at given time points after injection of glucose. **C,** quantification of baseline blood glucose, two-way ANOVA, data are represented as mean ± SEM. **D,** area under the curve (AUC) of blood glucose profiles during IPGTT. unpaired 2-tailed t-test. **E, F,** Insulin tolerance test performed on PHD2 ECKO and corresponding control mice. Glucose was measured from blood at given time points after injection of insulin. **G,** quantification of baseline blood glucose, unpaired 2-tailed t-test, data are represented as mean ± SEM. **H,** Area under the curve (AUC) of blood glucose profiles during ITT. unpaired 2-tailed t-test, data are represented as mean ± SEM, n = 6,8

Given the lean phenotype observed in PHD2 ECKO mice and the well-known association between obesity and insulin resistance, we further investigated the impact of PHD2 ECKO on glucose metabolism. In young and aged mice, PHD2 ECKO mice displayed lower glucose intolerance compared with control mice during the intraperitoneal glucose tolerance test (IPGTT) (Fig. 5A-D). Additionally, in the insulin tolerance test (ITT) (Fig. 5E-H), PHD2 ECKO mice demonstrated increased insulin sensitivity compared with controls. These findings indicate that a deficiency of PHD2 in endothelial cells has a positive effect on insulin sensitivity in both young and aged mice.

## Discussion

The present study has several major new findings. First, we found that vascular endothelial-specific mPHD2 gene KO (PHD2 ECKO) significantly attenuates age-related obesity without affecting lean mass gain. Second, aged PHD2 ECKO mice displayed a significantly increased exercise capacity as compared to aged PHD2^F/F^ mice. However, the exercise capacity was similar between young PHD2 ECKO mice and young PHD2^F/F^ mice. Third, we found that the daily energy expenditure was significantly increased with no significant changes in food intake in PHD2 ECKO mice. Furthermore, we found that glucose tolerance capacity and insulin sensitivity were significantly improved in both young and aged PHD2 ECKO mice.

One of the most interesting findings is that aged PHD2 ECKO mice exhibited significantly reduced obesity, characterized by reduced fat mass and its ratio to lean mass, tibial length, or body weight. The reduction of fat mass was accompanied by a decrease in adipocyte size in WAT. Aged PHD2 ECKO mice also exhibited higher oxygen consumption without reduction of food update as compared with control mice, suggesting the increased energy expenditure contributes to their leaner phenotyping. While the finding of reduced age-related obesity in vascular endothelial PHD2 deficient mice has not been previously reported, the effect of global PHD2 deficiency in attenuating obesity has been previously observed. Thus, using a HIF-p4h-2 hypomorph mouse line, previous studies also demonstrated that an increase of HIF stability by systemic inhibition of PHD2 significantly attenuated obesity and metabolic dysfunction [25]. A recent study from the same group further showed that HIF-p4h-2 hypomorph mice were resistant to aging-induced obesity and glucose intolerance [26]. In addition, a previous study also demonstrated that PHD2 deficiency in adipocytes significantly attenuated diet-induced obesity and glucose intolerance in mice [19, 27]. One of the potential mechanisms for the reduced obesity in the aged PHD2 ECKO mice might be due to the elevated energy expenditure by increased heat production in these mice. The increased heat generation in PHD2 ECKO mice is likely an adaptive response because extra heat generation might be needed to maintain the core body temperature for mice with lower subcutaneous adipose tissue to form an insulating barrier around the body.

Another interesting finding is that PHD2 ECKO resulted in a significant increase in exercise capacity in aged mice. PHD2 ECKO mice also had significantly higher oxygen consumption and VCO2 production under control conditions and during treadmill exercise in these mice. The increased oxygen consumption and VCO2 production were observed at both nighttime and daytime, indicating the systemic metabolic rate and energy expenditure are upregulated in these PHD2 ECKO mice. The potential mechanisms underlying the enhanced exercise capacity and energy expenditure in these mice likely involve, but are not limited to, the activation of HIF1 and HIF2 signaling pathways. First, PHD2 deficiency can enhance angiogenesis, vessel remodeling/generation, and collateral vessel formation by increasing HIF1 stability in skeletal and cardiac muscles partially through modulating HIF1, VEGF, and NO signaling pathways [28, 29]. Indeed, studies showed that inducible PHD2 deficiency led to hyperactive angiogenesis in the heart and other tissues [30]. In addition, PHD2 deficiency increases erythropoietin (EPO) production [20, 30, 31], while EPO can improve exercise performance through its interaction with EPO receptors broadly distributed in both hematopoietic and non-hematopoietic tissues [32]. For example, EPO stimulates red blood cell production to improve oxygen transportation during exercise. EPO also stimulates skeletal muscle mitochondrial biogenesis gene expression (such as PGC-1α) and skeletal muscle fiber programming to type I muscle fibers [33]. In addition, EPO attenuates diet-induced obesity, improves glucose tolerance, reduces insulin resistance, and attenuates fat mass accumulation [32, 34]. Lastly, the reduced fat mass and body weight in aged PHD2 ECKO mice can certainly decrease work load and directly enhance exercise capacity.

While systemic and endothelial-specific PHD2 inhibition attenuates obesity and metabolic dysfunction in mice, we also noticed that several aged PHD2 ECKO mice died during the study, indicating a detrimental effect of endothelial-specific PHD2 inhibition on the life span. In addition, several previous studies have demonstrated that both systemic and endothelial-specific PHD2 inhibition resulted in severe diseases such as idiopathic pulmonary hypertension and congestive heart failure in mice [35, 36]. For example, Tie2^Cre^-mediated PHD2 deletion caused a spontaneous severe PAH, as evidenced by extensive pulmonary vascular remodeling, vascular occlusion, plexiform-like lesions, and right ventricular (RV) hypertrophy and failure [36]. The above complex pathological and molecular changes were mainly attributed to the activation of HIF-2α after PHD2 deletion [36]. In addition, CDH5^Cre^-mediated PHD2 deletion also caused a mild age-dependent PAH in mice [37]. A recent study further demonstrated that Tie2^Cre^-mediated PHD2 deletion caused LV hypertrophy and dysfunction through HIF2a dependent pathway [35]. Furthermore, global PHD2 deficiency leads to erythrocytosis and thrombosis formation by enhancing EPO production [20]. Moreover, conditional inactivation of PHD2 (using chicken beta-actin-Cre-ER mice together with tamoxifen injection) resulted in an increase in red blood cell production, venous congestion, and dilated cardiomyopathy in mice [38]. The increased mortality in aged male PHD2 ECKO mice also indicate a detrimental effect of PHD2 inhibition on life span in mice. Thus, treating obesity and metabolic dysfunction by either systemic or endothelial-specific PHD2 inhibition may cause unwanted side effects such as cardiac dysfunction and increased mortality.

Overall, the findings of this study highlight the importance of endothelial PHD2 in regulating metabolic status and maintaining metabolic homeostasis, particularly during aging. While the absence of PHD2 in endothelial cells appears to confer apparent metabolic benefits against obesity, systemic or endothelial-specific PHD2 deficiency also causes detrimental effects in the cardiopulmonary system. Thus, caution should be taken to explore inhibition of PHD2 as a potential therapeutic target for addressing age-related metabolic abnormalities and obesity-related disorders. In the context that sex is an important biological factor that affects obesity, glucose sensitivity, and exercise capacity, there is a possibility that female and male mice may have different changes of obesity and exercise capacity after endothelial-specific PHD2 deficiency. Thus, studying only male mice is a major limitation of our study. Further research is clearly needed to fully understand any potential sex differences, the underlying mechanisms, and the potential clinical implications of targeting PHD2 in the context of metabolic health.

### Supplementary Information

Below is the link to the electronic supplementary material.

Supplementary file1 (DOCX 2287 KB)

## Data Availability

Data will available when it is requested.
