# Supplementary material for: Endothelial specific prolyl hydroxylase domain-containing protein 2 deficiency attenuates aging-related obesity and exercise intolerance
Source: GeroScience. 2024 Mar 11;46(4):3945–56. doi: 10.1007/s11357-024-01108-0 (PMC11226575; doi:10.1007/s11357-024-01108-0)
Supplement: Supplementary file 1 — Supplementary file1 (DOCX 2287 KB) [file 11357_2024_1108_MOESM1_ESM.docx]

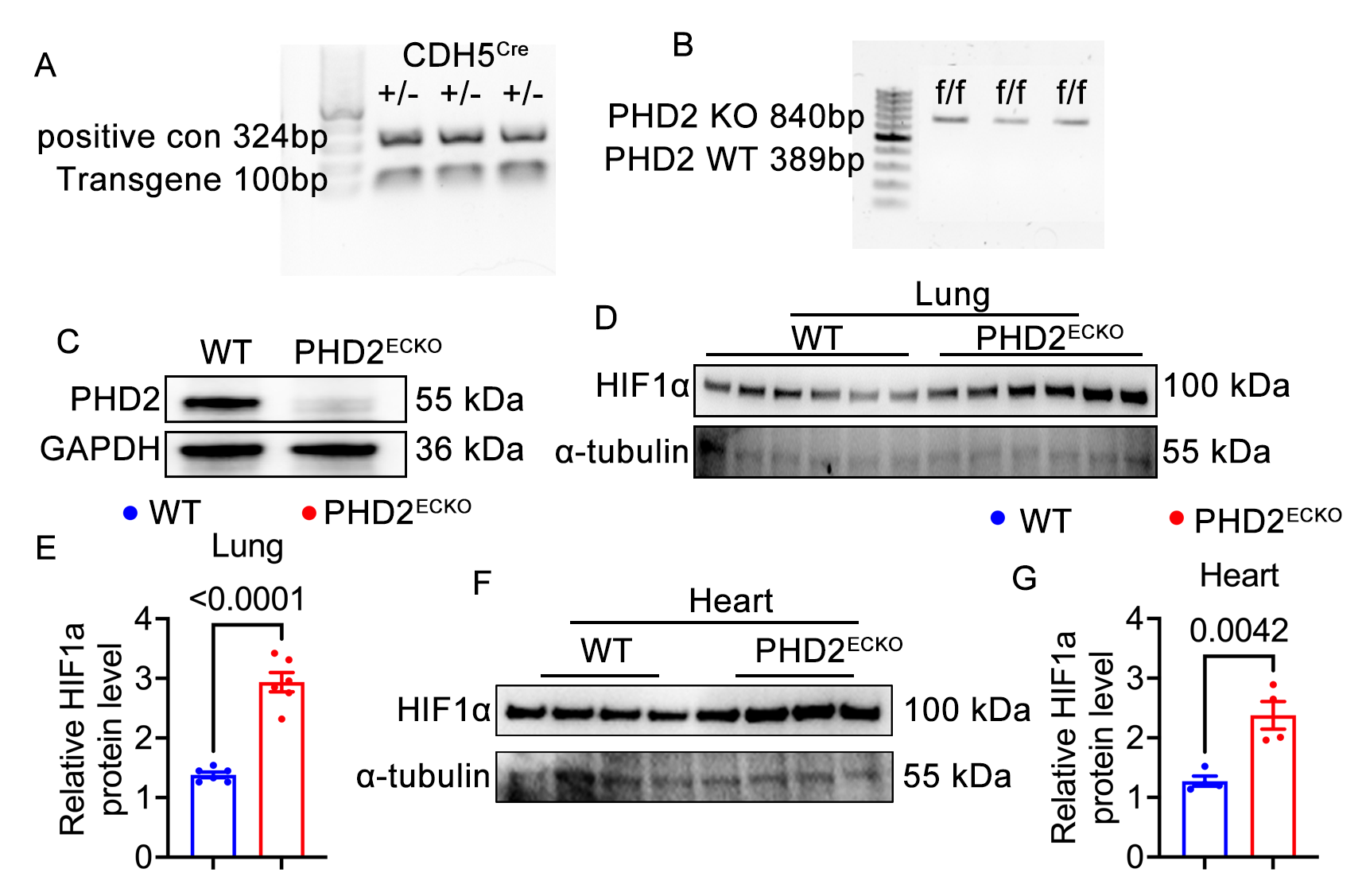


Supplemental Figure 1. Genotyping of PHD2 ECKO mice (**A, B**). Western blot analysis of PHD2 deficiency in endothelial cells (**C**). HIF1α level in Lungs of WT and PHD2 ECKO mice (**D, E**). HIF1α level in hearts of WT and PHD2 ECKO mice (**F, G**).


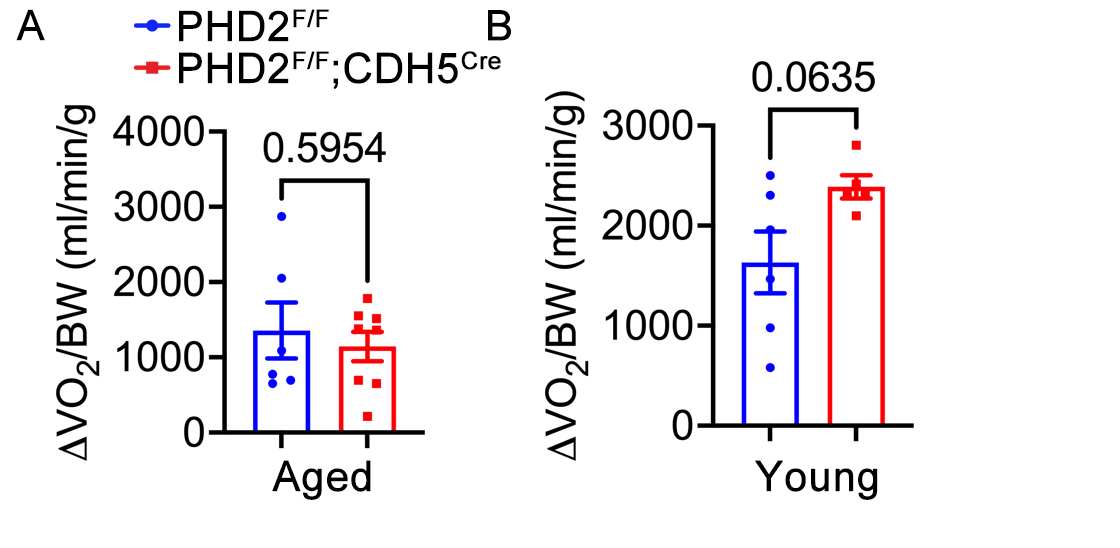


Supplemental Figure 2. PHD2 ECKO mice and control groups were run on the treadmill and their metabolism and maximum oxygen consumption minus baseline oxygen were recorded. **A,** ΔVO_2_ consumption in aged mice. **B,** ΔVO_2_ consumption in young mice
